# Phenology of *Lippia lasiocalycina* Cham.: A Wild Medicinal Species Native to Brazil

**DOI:** 10.3390/plants15182738

**Published:** 2026-09-08

**Authors:** Maria Clara de Almeida Lima Rocha, Lenaldo Muniz de Oliveira, Mariluci Lopes Gonzaga

**Affiliations:** 1 Postgraduate Program in Plant Genetic Resources (PPGRGV), Department of Biological Sciences (DCBIO), Feira de Santana State University (UEFS), Feira de Santana 44036-900, BA, Brazil; marilucigonzaga@gmail.com; 2 Department of Biological Sciences (DCBIO), Feira de Santana State University (UEFS), Feira de Santana 44036-900, BA, Brazil

**Keywords:** phenophases, native medicinal plants, Lamiaceae, cultivation, plant genetic resources

## Abstract

*Lippia lasiocalycina* Cham. is a wild medicinal species native to Brazil, belonging to the Lamiaceae family, with bioactive potential in its essential oils. However, it remains poorly studied, especially in the fields of conservation and domestication. Phenological studies enable the understanding of relationships between abiotic factors and plant developmental stages, supporting cultivation strategies. In this context, the objective of this study was to evaluate the effect of seasonality on the phenology of *Lippia lasiocalycina* under the environmental conditions of Feira de Santana, Bahia. The species was propagated and cultivated under field conditions, and phenological data were collected biweekly over 12 months to assess the intensity and synchrony of phenophases. Climatic variables were obtained from specialized sources and correlated with phenological events, including circular statistical analysis. The species showed high phenological synchrony, with continuous budburst and mature leaves throughout the year, indicating potential for year-round exploitation. Fruiting, however, exhibited seasonal behavior and a negative correlation with precipitation. Leaf fall was negatively correlated with precipitation and humidity, and positively associated with radiation, temperature, and photoperiod, while senescence showed the opposite trend. These results contribute to the development of cultivation strategies and to defining optimal periods for management and harvesting.

## 1. Introduction

Brazil harbors one of the greatest plant biodiversities in the world, with species distributed across its territory in environments with distinct climatic characteristics [1]. Within this diversity, there is a high number of medicinal plant species, both currently used and with potential applications. The semi-arid region stands out due to the interaction between plants and the region’s high temperatures and water deficit, and it hosts a considerable number of taxa rich in bioactive compounds with potential use by industry [2]. The genus *Lippia* Linn. comprises around 200 species, predominantly distributed in the Brazilian semi-arid region [3]. This genus has been extensively studied due to the medicinal uses of its species, as the essential oils extracted from them exhibit significant biological activity against fungi, bacteria, insects, and others. These oils represent an important source of raw material for the food, pharmaceutical, cosmetic, agrochemical, and related industries [4,5].

The species *Lippia lasiocalycina* Cham. is a wild shrub native to Brazil [3], capable of reaching approximately two meters in height, with simple, opposite, oval-shaped leaves and tubular flowers with pink coloration and a yellow-and-white interior. Studies have reported fungicidal and insecticidal activity of its essential oils [6,7,8]. However, this species remains poorly studied, particularly regarding conservation and domestication. As a non-domesticated species subject to uncontrolled extractivism, research in these areas is essential.

In this context, several studies have evaluated the interaction between the environment and the phenological stages of plants, demonstrating that environmental conditions influence vegetation dynamics, growth, and reproduction [9]. Thus, it is possible to correlate abiotic factors with plant phenophases and identify their interactions [10,11], enabling the observation and understanding of the plant’s characteristic phases, an essential step for its conservation and domestication. This, in turn, supports decision-making in the production process, such as determining the best timing for harvesting, propagation, irrigation, fertilization, pruning, and other practices [12].

The literature includes studies on various *Lippia* species, such as *Lippia sidoides* Cham., which exhibits high synchrony in fruiting and leaf fall, with sprouting, mature leaves, flowering, and leaf abscission strongly influenced by abiotic factors, especially precipitation and air humidity [13]. For *Lippia lupulina* Cham., fruit maturity has been reported at the end of the dry season, with a peak in dispersal at the beginning of this period [14]. For *Lippia dulcis* Trevir, increased flowering and fruiting have been observed during periods of low precipitation [15]. However, for *L. lasiocalycina*, no phenological studies or correlations between phenophases and environmental conditions have been reported to date.

Therefore, this study aimed to evaluate the effect of seasonality on the phenology of *Lippia lasiocalycina* Cham. under the environmental conditions of Feira de Santana, Bahia, thereby contributing to research in the areas of species conservation and domestication.

## 2. Results

### 2.1. Climatic Characterization

The highest precipitation levels were recorded in January (147.2 mm) and February (143.2 mm), while the lowest levels occurred in November (20.8 mm) and December (5.2 mm). Relative humidity increased in the initial months, peaked on 30 April, and decreased from June onwards, reaching its lowest levels in December.

The lowest average temperatures were observed in July (22.89 °C and 22.68 °C), while the highest temperatures occurred in December (28.06 °C and 28.85 °C). The photoperiod decreased to 11 h between 15 June and 15 July and reached its maximum in December (12:49:16 h and 12:50:15 h).

**Figure 1:**
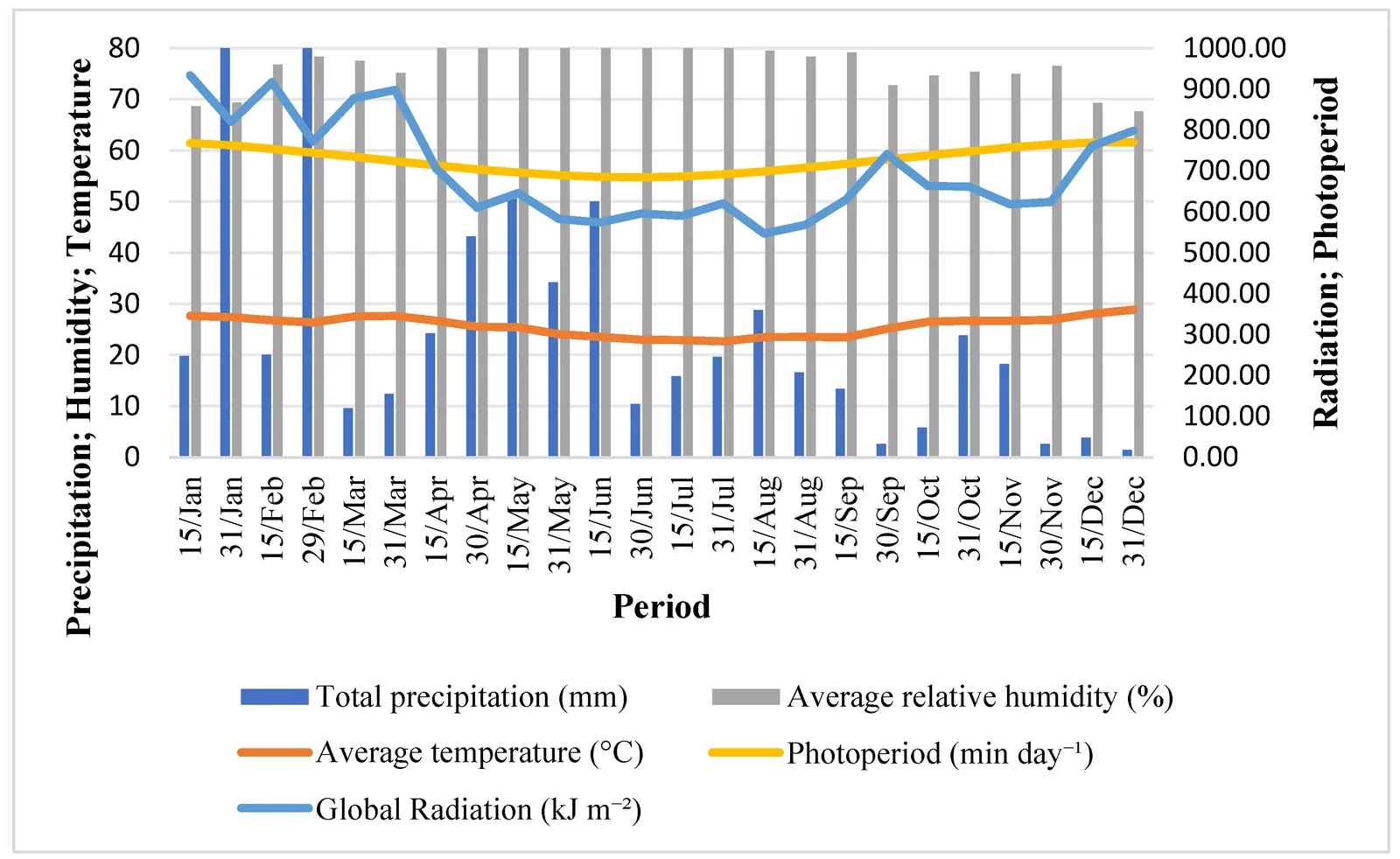
Total precipitation data (mm), average relative humidity (%), average temperature (°C), photoperiod (min day^−1^), and global radiation (KJ m^−2^) under the conditions of Feira de Santana, Bahia, Brazil, from January to December 2024.

Solar radiation reached its highest value on 15 January (933.63 KJ·m^−2^) and the lowest on 15 August (546.88 KJ·m^−2^) (Figure 1).

### 2.2. Circular Statistics of Phenophases

Circular statistical analysis indicated that the budding and mature leaves phenophases had no defined mean angle or mean date, occurring continuously throughout the year, with vector lengths close to zero, indicating a uniform temporal distribution (Table 1).

Senescence, flowering, and leaf fall showed low temporal concentration (low r values) and non-significant Rayleigh tests (*p* > 0.05), indicating absence of a defined seasonal pattern, despite mean dates occurring around 20 May, 10 June, 5 and January, respectively.

Fruiting showed higher temporal concentration than the other phenophases and a significant Rayleigh test result (*p* < 0.05), indicating seasonal distribution throughout the annual cycle (Table 1).

### 2.3. Population Activity Index

**Figure 2:**
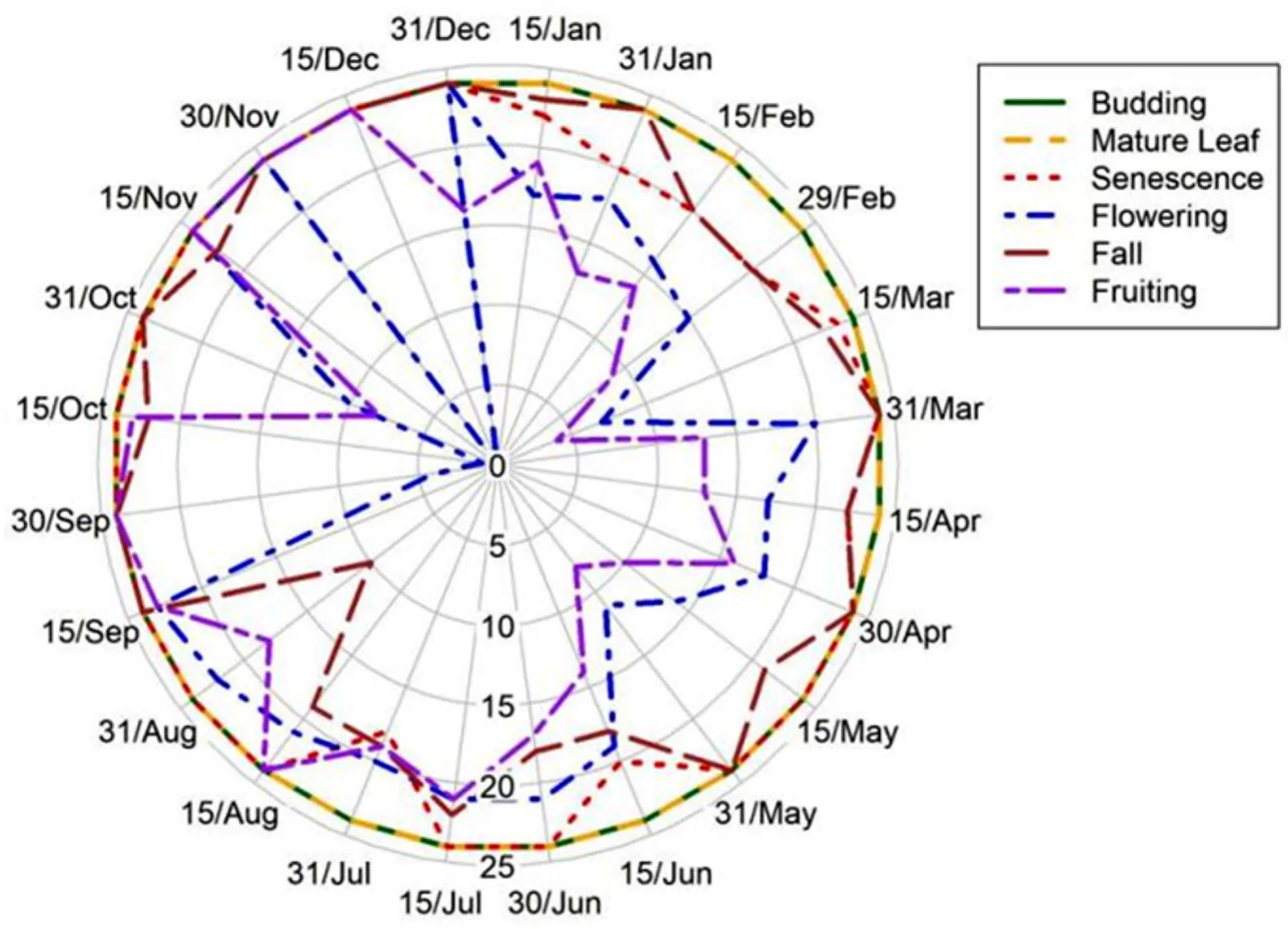
Population activity index of the phenophases of budding, leaf fall, mature leaves, leaf senescence, flowering, and fruiting in *Lippia lasiocalycina* Cham. from January to December 2024 under the environmental conditions of Feira de Santana, Bahia, Brazil. The radial axis represents the number of individuals expressing each phenophase during each evaluation period.

More than 60% of individuals exhibited the same phenophase simultaneously, indicating a high activity index (Figure 2).

Budding and mature leaves showed a 100% activity index throughout the entire period. Senescence and leaf fall presented peaks at different times of the year.

Flowering reached a 100% activity index in November and on 31 December, with an absence of flowers during the first half of December. Fruiting showed peaks on 15 August and between 15 November and 15 December, with a lower occurrence on 15 March.

### 2.4. Intensity of Phenophases

**Figure 3:**
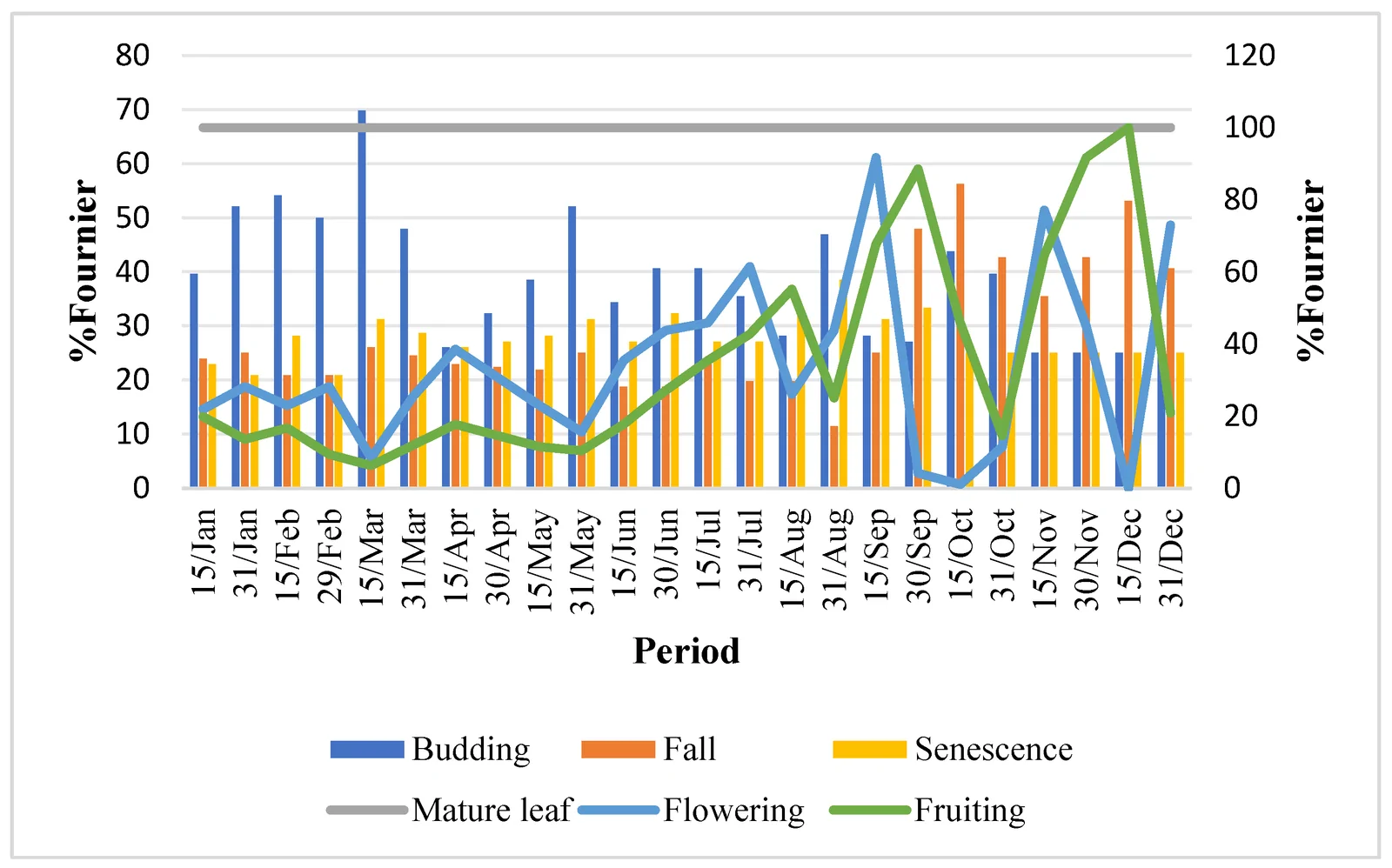
Intensity of events according to the Fournier method for the phenophases of budding, leaf fall, mature leaves, leaf senescence, flowering, and fruiting of *Lippia lasiocalycina* Cham. cultivated under the conditions of Feira de Santana, Bahia, Brazil, from January to December 2024. The left *y*-axis represents budding, leaf fall, and senescence, whereas the right *y*-axis represents mature leaves, flowering, and fruiting.

Mature leaves showed 100% intensity throughout all evaluation periods (Figure 3).

Senescence ranged from 20.83% (31 January) to 38.54% (15 August). Budding showed a maximum intensity of 69.79% on 15 March.

Flowering reached 91.67% on 15 September and was absent on 15 December. Fruiting varied from 6.25% (15 March) to 100% (15 December).

Leaf fall reached its lowest value on 31 August (11.46%) and increased toward the end of the year.

Most phenophases showed intensity values between 26% and 50% across most evaluation periods, with some reaching values between 51% and 100%.

### 2.5. Correlation Between Phenophases and Climatic Variables

**Figure 4:**
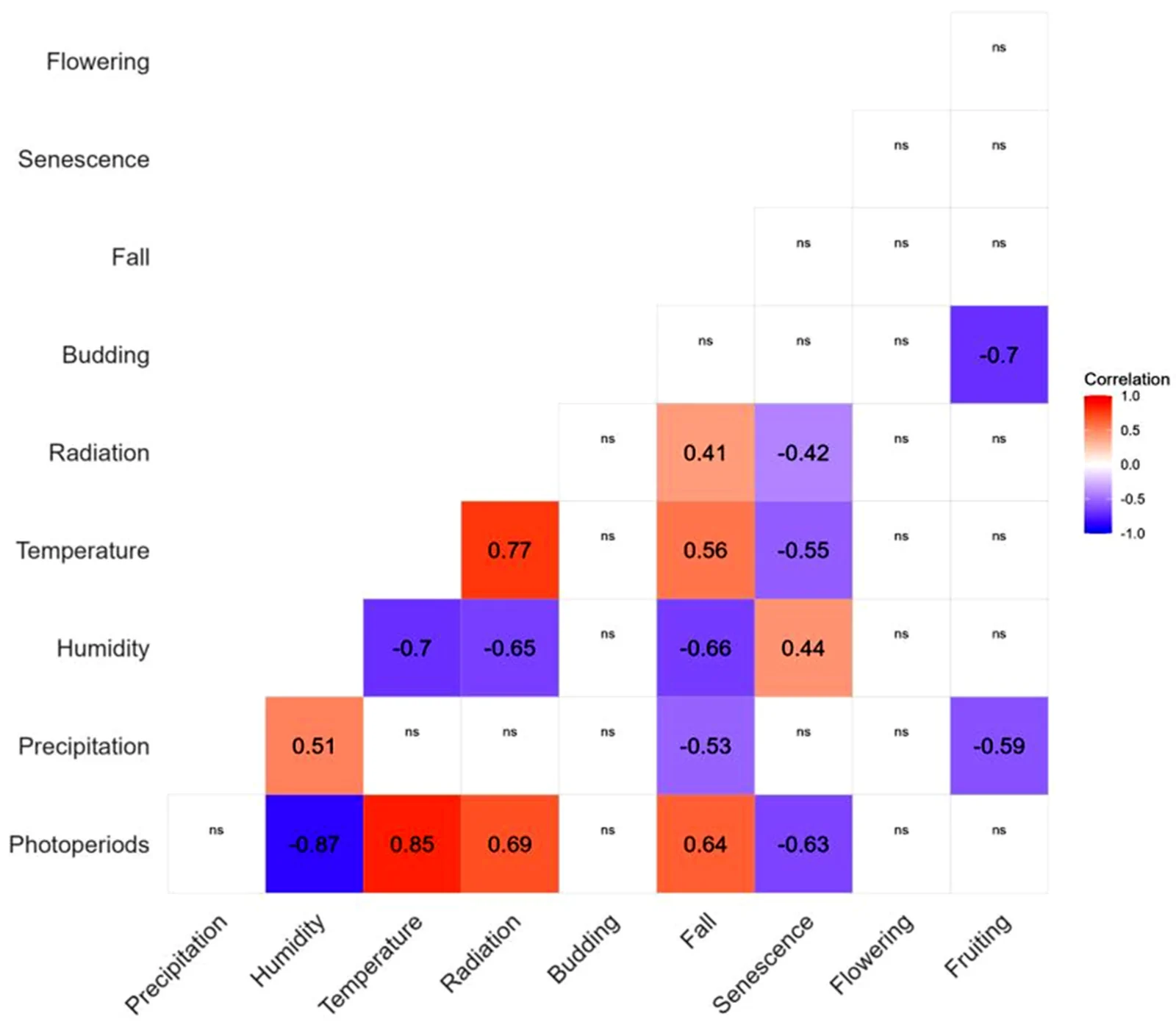
Spearman correlation coefficients between climatic variables and the phenophases of budding, leaf fall, leaf senescence, flowering, and fruiting of the species *Lippia lasiocalycina* Cham. cultivated under the environmental conditions of Feira de Santana, Bahia, Brazil, from January to December 2024. ns = Non-significant correlation (*p* > 0.05).

Significant correlations were observed between radiation, temperature, humidity, and photoperiod with leaf fall and senescence, as well as between precipitation with leaf fall and fruiting (Figure 4).

**Figure 5:**
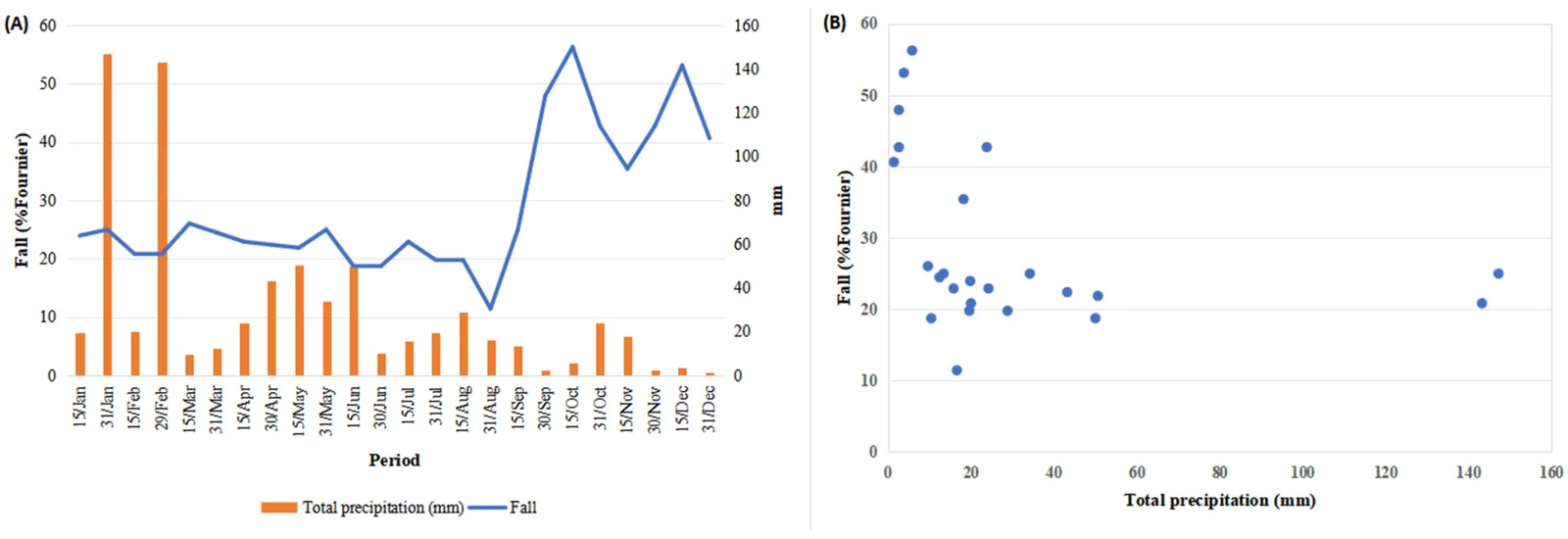
(**A**) Temporal variation in leaf fall (% Fournier) and total precipitation (mm) of *Lippia lasiocalycina* Cham. cultivated under the conditions of Feira de Santana, Bahia, Brazil, from January to December 2024. (**B**) Relationship between total precipitation (mm) and leaf fall (% Fournier), according to Spearman’s correlation analysis (ρ = −0.53) of the species *Lippia lasiocalycina* Cham.

**Figure 6:**
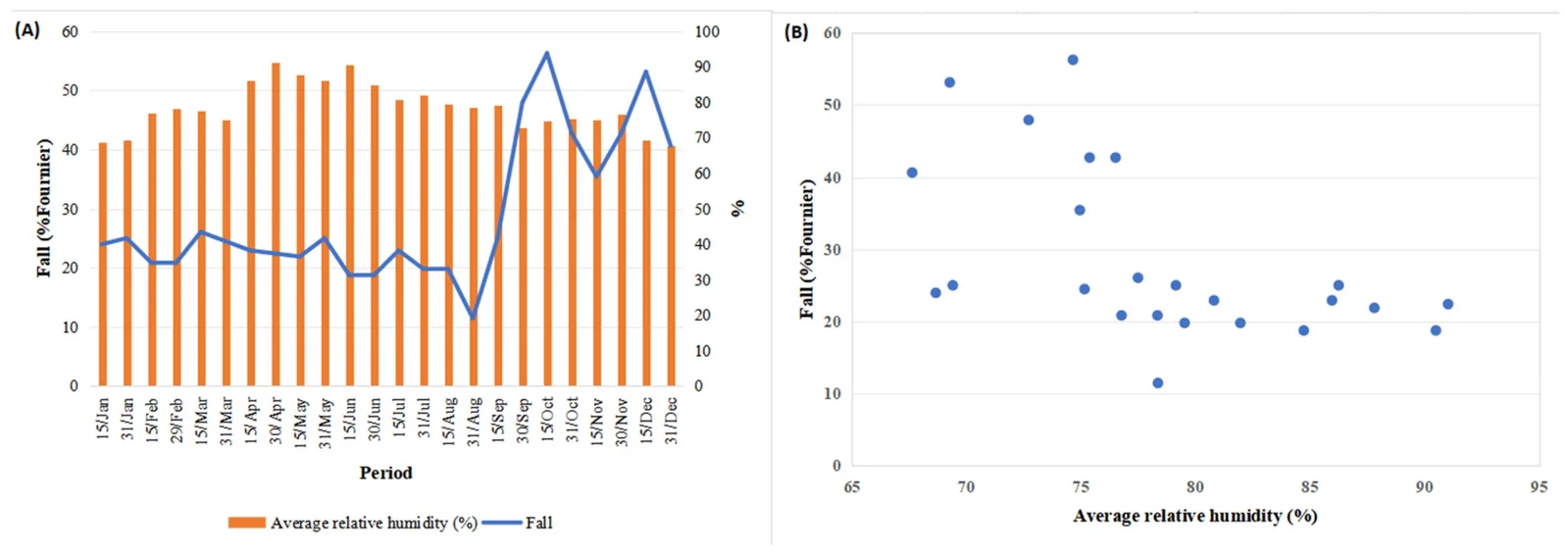
(**A**) Temporal variation in leaf fall (% Fournier) and average relative humidity (%) of *Lippia lasiocalycina* Cham. cultivated under the conditions of Feira de Santana, Bahia, Brazil, from January to December 2024. (**B**) Relationship between average relative humidity (%) and leaf fall (% Fournier) according to Spearman’s correlation analysis (ρ = −0.66) of the species *Lippia lasiocalycina* Cham.

Leaf fall showed moderate negative correlations with precipitation and relative humidity (Figure 5 and Figure 6).

**Figure 7:**
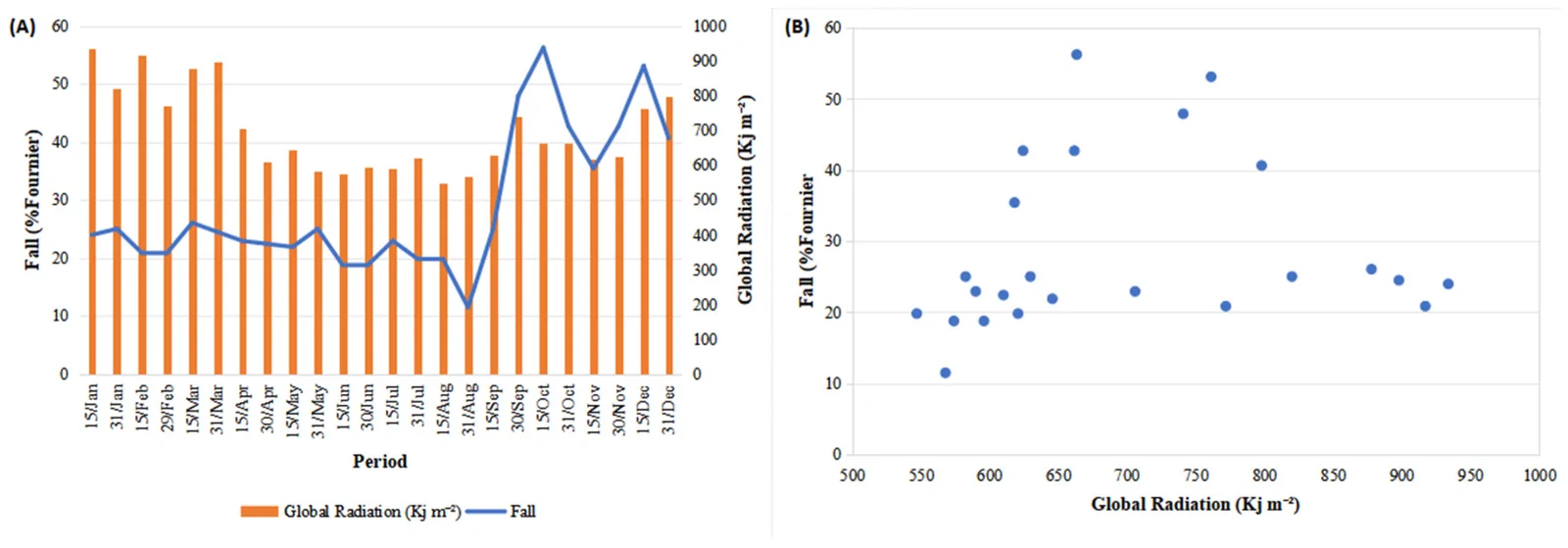
(**A**) Temporal variation in leaf fall (% Fournier) and global radiation (kJ m^−2^) of *Lippia lasiocalycina* Cham. cultivated under the conditions of Feira de Santana, Bahia, Brazil, from January to December 2024. (**B**) Relationship between global radiation (kJ m^−2^) and leaf fall (% Fournier) according to Spearman’s correlation analysis (ρ = 0.41) of the species *Lippia lasiocalycina* Cham.

**Figure 8:**
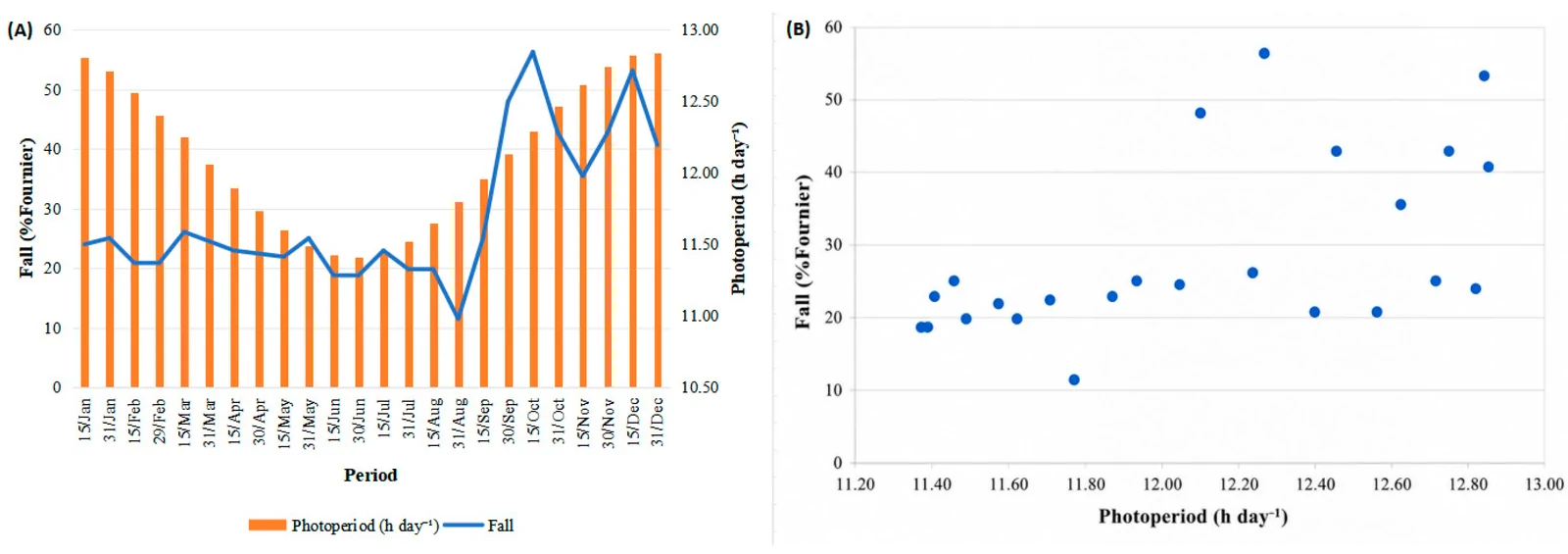
(**A**) Temporal variation in leaf fall (% Fournier) and photoperiod (h day^−1^) of *Lippia lasiocalycina* Cham. cultivated under the conditions of Feira de Santana, Bahia, Brazil, from January to December 2024. (**B**) Relationship between photoperiod (h day^−1^) and leaf fall (% Fournier) according to Spearman’s correlation analysis (ρ = 0.64) of the species *Lippia lasiocalycina* Cham.

**Figure 9:**
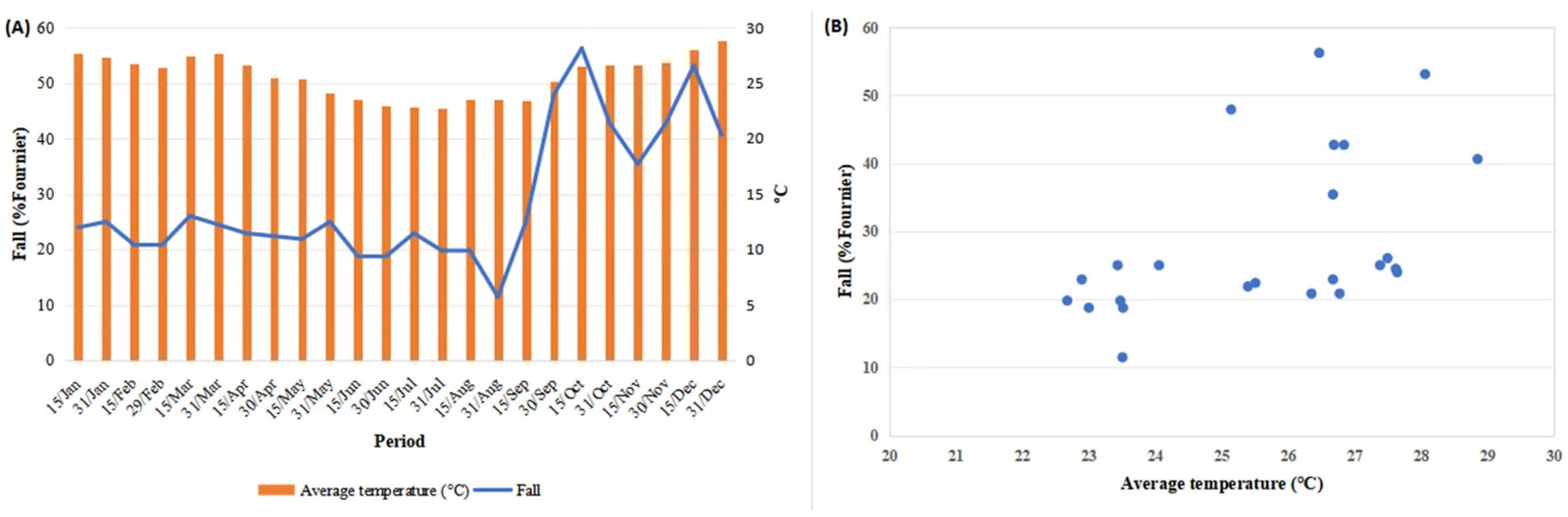
(**A**) Temporal variation in leaf fall (% Fournier) and average temperature (°C) of *Lippia lasiocalycina* Cham. cultivated under the conditions of Feira de Santana, Bahia, Brazil, from January to December 2024. (**B**) Relationship between average temperature (°C) and leaf fall (% Fournier) according to Spearman’s correlation analysis (ρ = 0.56) of the species *Lippia lasiocalycina* Cham.

Leaf fall showed moderate positive correlations with global radiation, temperature, and photoperiod (Figure 7, Figure 8 and Figure 9).

An inverse relationship between senescence and leaf fall was observed.

**Figure 10:**
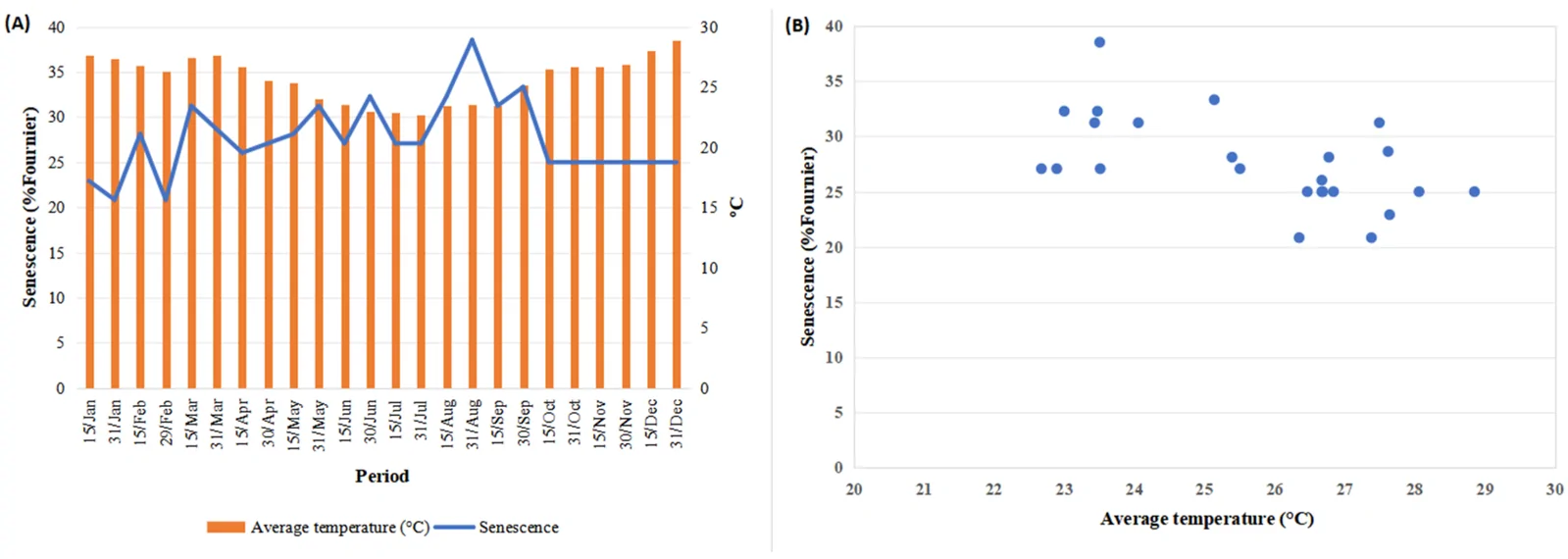
(**A**) Temporal variation in senescence (% Fournier) and average temperature (°C) of *Lippia lasiocalycina* Cham. cultivated under the conditions of Feira de Santana, Bahia, Brazil, from January to December 2024. (**B**) Relationship between average temperature (°C) and senescence (% Fournier) according to Spearman’s correlation analysis (ρ = −0.55) of the species *Lippia lasiocalycina* Cham.

**Figure 11:**
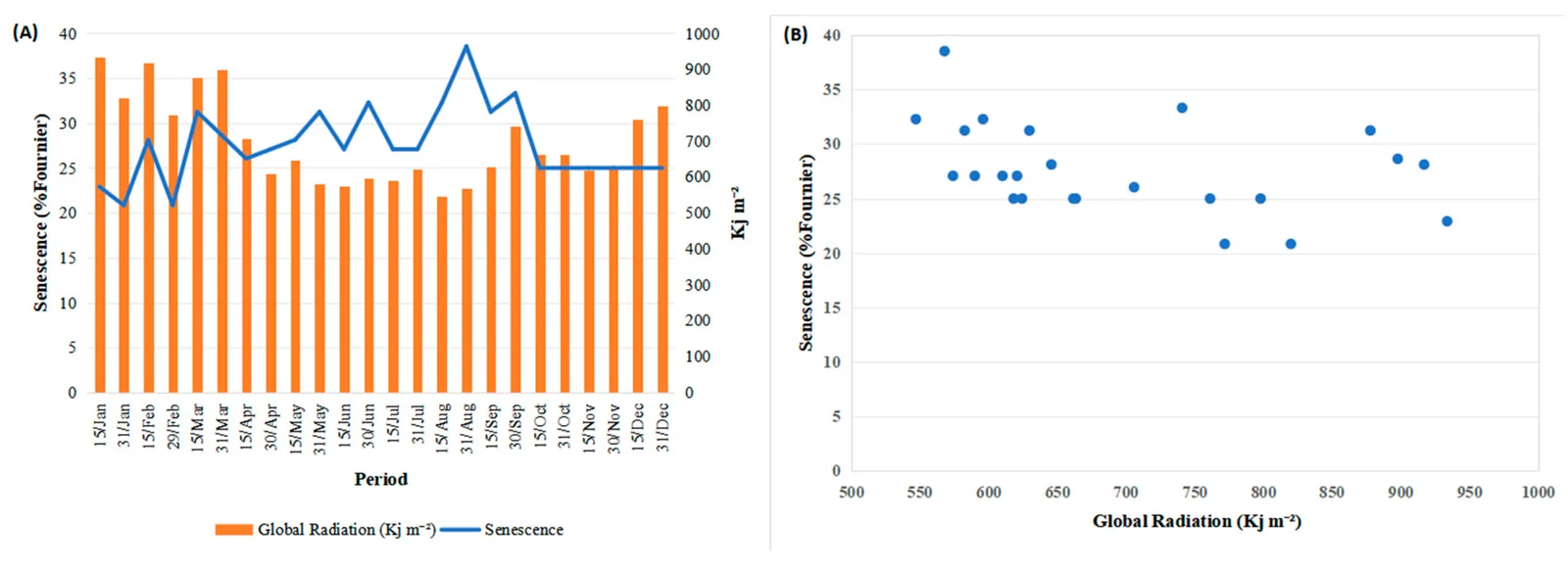
(**A**) Temporal variation in senescence (% Fournier) and global radiation (kJ m^−2^) of *Lippia lasiocalycina* Cham. cultivated under the conditions of Feira de Santana, Bahia, Brazil, from January to December 2024. (**B**) Relationship between global radiation (kJ m^−2^) and senescence (% Fournier) according to Spearman’s correlation analysis (ρ = −0.42) of the species *Lippia lasiocalycina* Cham.

**Figure 12:**
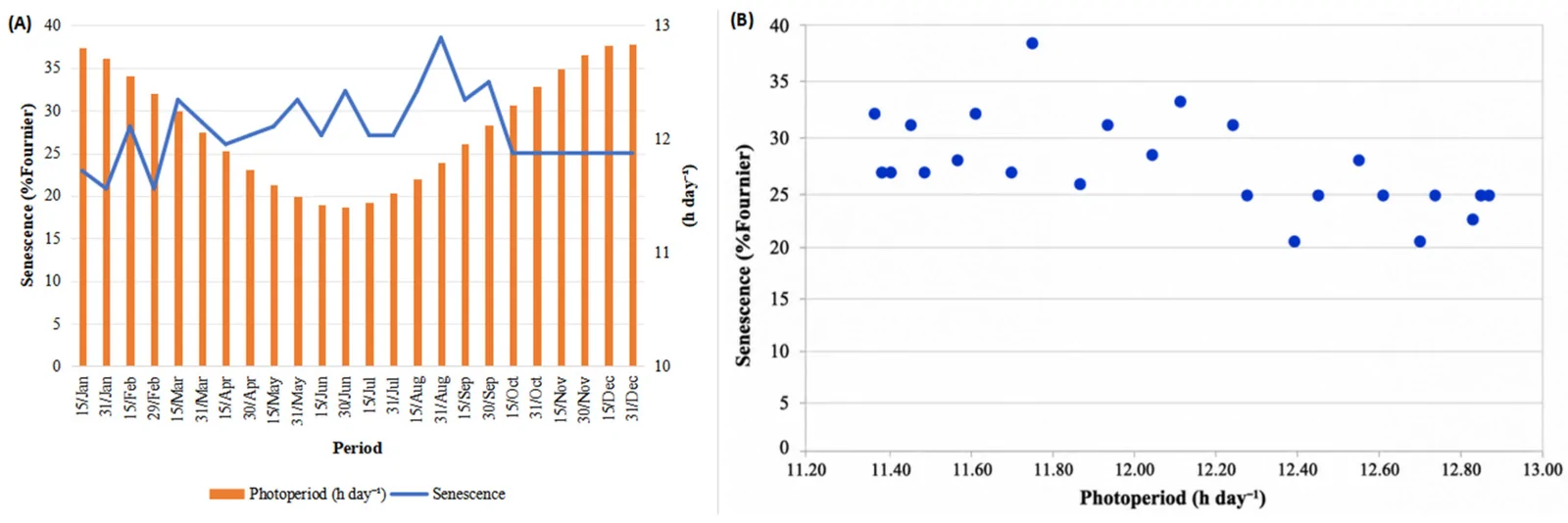
(**A**) Temporal variation in senescence (% Fournier) and photoperiod (h day^−1^) of *Lippia lasiocalycina* Cham. cultivated under the conditions of Feira de Santana, Bahia, Brazil, from January to December 2024. (**B**) Relationship between photoperiod (h day^−1^) and senescence (% Fournier) according to Spearman’s correlation analysis (ρ = −0.63) of the species *Lippia lasiocalycina* Cham.

Senescence showed moderate negative correlations with temperature, global radiation and photoperiod (Figure 10, Figure 11 and Figure 12).

**Figure 13:**
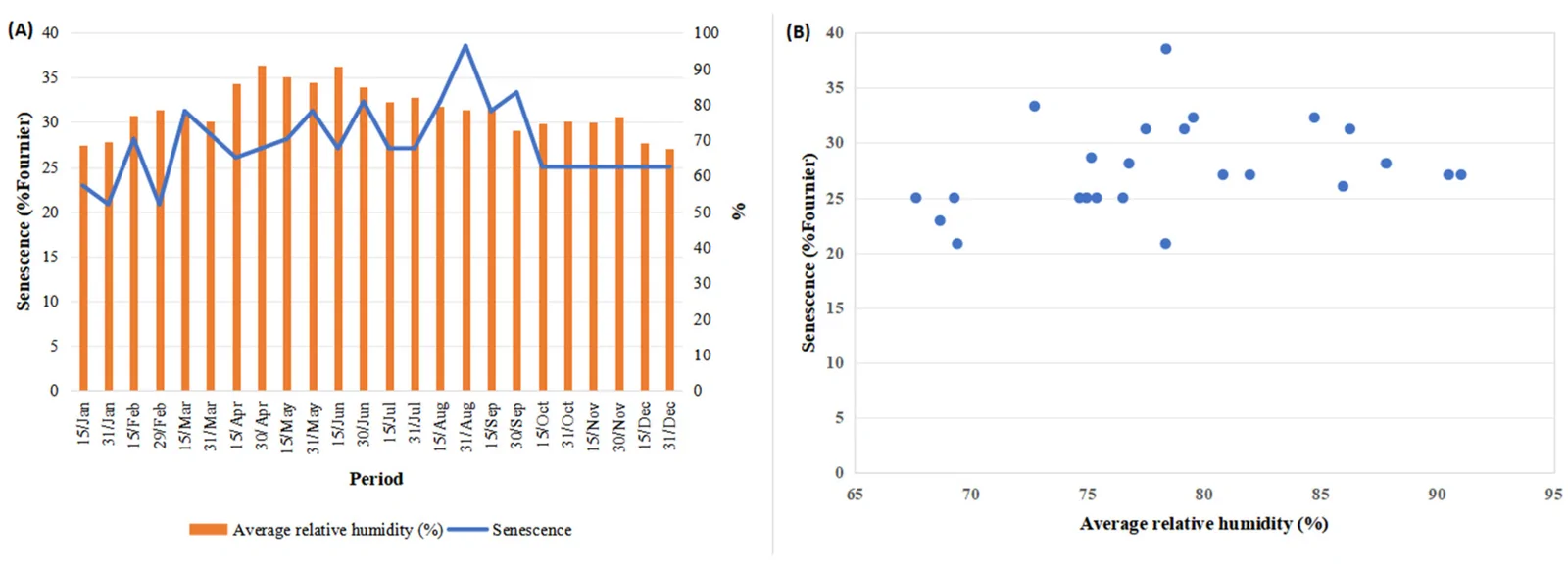
(**A**) Temporal variation in senescence (% Fournier) and average relative humidity (%) of *Lippia lasiocalycina* Cham. cultivated under the conditions of Feira de Santana, Bahia, Brazil, from January to December 2024. (**B**) Relationship between average relative humidity (%) and senescence (% Fournier) according to Spearman’s correlation analysis (ρ = 0.44) of the species *Lippia lasiocalycina* Cham.

Senescence showed a moderate positive correlation with relative humidity (Figure 13).

**Figure 14:**
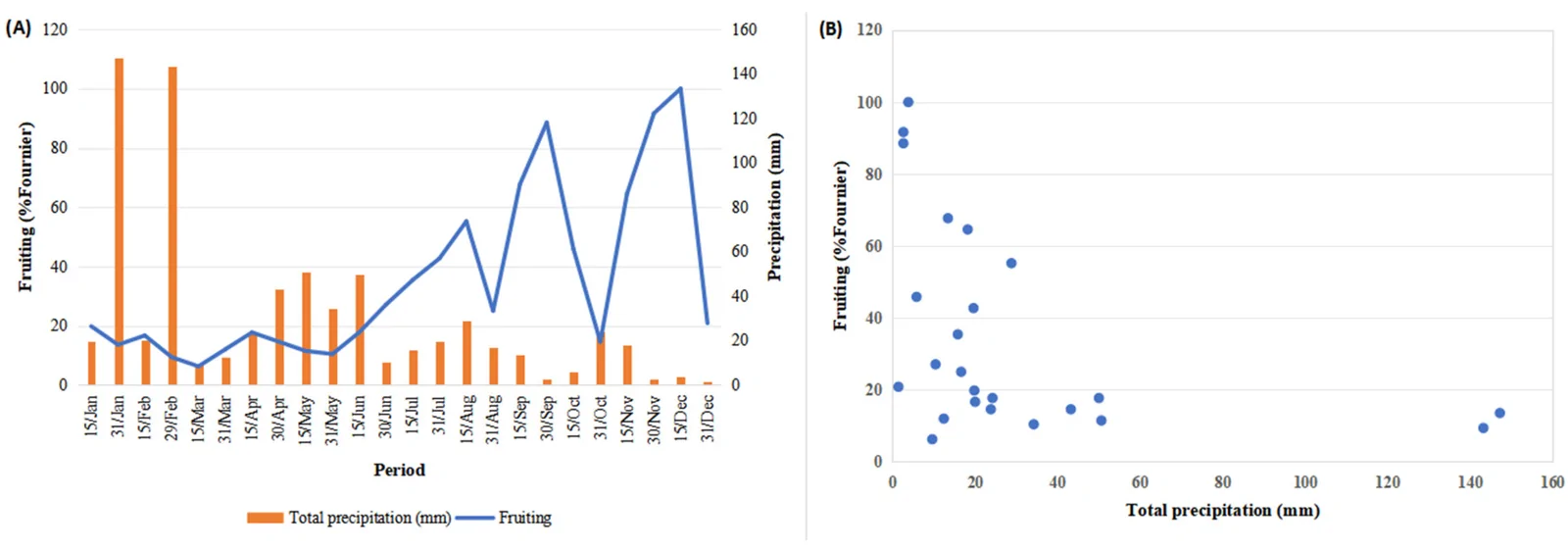
(**A**) Temporal variation in fruiting (% Fournier) and total precipitation (mm) of *Lippia lasiocalycina* Cham. cultivated under the conditions of Feira de Santana, Bahia, Brazil, from January to December 2024. (**B**) Relationship between total precipitation (mm) and fruiting (% Fournier) according to Spearman’s correlation analysis (ρ = −0.59) of the species *Lippia lasiocalycina* Cham.

Fruiting showed a moderate negative correlation with precipitation (Figure 14).

## 3. Discussion

The observed patterns indicate that *Lippia lasiocalycina* exhibits a dynamic phenological behavior influenced by environmental conditions. The high phenological synchrony [16] among individuals may be advantageous, as it can reduce the effects of herbivory and ensure that phenophases occur under more favorable conditions [17], in addition to supporting the planning of cultivation and harvesting strategies. Although a high proportion of individuals simultaneously expressed some phenophases according to the activity index, circular statistics indicated low temporal concentration of events throughout the year, suggesting that the species does not strictly depend on specific environmental windows for the occurrence of most events. The continuous occurrence of budding and mature leaves throughout the year indicates persistent vegetative activity under the environmental conditions of the study area. Fruiting showed greater temporal concentration compared to the other phenophases, with a significant Rayleigh test (*p* < 0.05), indicating seasonal distribution throughout the annual cycle.

The absence of a well-defined seasonality for budding, mature leaves, flowering, senescence, and leaf fall suggests that these phenophases may occur continuously throughout the year. This pattern reflects a flexible phenological strategy, possibly associated with the environmental conditions of the study area. In contrast, fruiting exhibited a more clearly defined seasonal pattern, indicating greater sensitivity of this phenophase to climatic variations. The apparent inversion between the mean dates of flowering and fruiting likely reflects the overlap of phenophases throughout the year and their low concentration, rather than a true biological inversion of events.

The intensity data further reinforce the perennial nature of the species, with continuous production and maintenance of leaves throughout the year. This characteristic is particularly relevant, considering that leaves constitute the primary raw material for essential oil extraction. Despite this continuity, and the occurrence of peaks over the year, variations in the intensity of phenophases were observed, with most values ranging between 26% and 50%, which is considered low [18], although some phenophases occasionally reached values close to or equal to 100%. This allows for the development of more efficient management and harvesting strategies and contributes to more precise decision-making, particularly enabling leaf harvest in the second half of August, when leaf fall is minimal, while seed collection can be carried out between 30 November and 15 December, when fruiting was most intense.

The positive correlation between leaf fall and radiation, mean temperature, and photoperiod suggests that periods characterized by higher radiation, temperature, and photoperiod are associated with increased leaf fall. This pattern may reflect adaptive responses aimed at reducing water loss, leading to leaf abscission. Similarly, excessive light may cause photoinhibition, generating oxidative damage that accelerates leaf abscission, while an excessively long photoperiod may accelerate the plant’s life cycle [19]. In this context, a similar correlation has been reported for *Anadenanthera macrocarpa* (Benth.) [20]. Furthermore, increased temperature has been shown to promote leaf fall in *Cordia oncocalyx* Allemann and *Poincianella pyramidalis* (Tul.) L.P. Queiroz [21].

On the other hand, the negative correlation between leaf fall and precipitation and humidity indicates the existence of a species-specific mechanism to reduce the number of leaves under conditions of low water availability in order to minimize transpiration, conserve energy allocated to photosynthesis, and remobilize nutrients for the production of new organs [22,23]. This pattern is consistent with studies that, when evaluating the influence of precipitation on Caatinga vegetation, observed that water availability during the rainy season significantly increases biomass, whereas reduced rainfall leads to a decrease in biomass, altering the landscape [9]. Additionally, greater leaf loss under low-precipitation conditions has been reported when correlating environmental variables with phenophases of *Elaeis guineensis* Jacq. [10], as well as an increase in leaf fall associated with soil water deficit in jabuticaba trees [11].

Leaf senescence showed an opposite pattern, with negative correlations between photoperiod, radiation, and temperature. Photoperiod refers to the duration of light exposure available for photosynthesis. Thus, when plants are exposed to a longer photoperiod, but still within their ideal range, they can maintain more consistent photosynthetic activity, delaying senescence. When combined with adequate radiation and temperature, plants exhibit increased photosynthetic and hormonal activity, postponing the need for premature senescence [19]. Similar results were observed in phenological studies of *Galianthe palustris* (Cham. & Schltdl.) Cabaña Fader & E.L. Cabral and *Stachytarpheta cayennensis* (Rich.) Vahl, in which negative correlations were found between senescence and photoperiod, and between senescence and temperature, respectively [24,25]. Comparable patterns were also reported for *Byrsonima pachyphylla* A. Juss. and *Byrsonima verbascifolia* (L.) DC. [26].

The positive correlation between senescence and relative humidity may reflect indirect effects of seasonal climatic variation rather than a direct physiological effect of humidity on leaf aging. Nevertheless, previous studies have suggested that increased humidity may contribute to reduced photosynthetic rates by limiting gas exchange, particularly CO_2_ uptake, which could potentially promote higher levels of senescence [19]. A similar pattern has been reported for *Elaeis guineensis* Jacq. [10].

The inverse relationship observed between senescence and leaf fall suggests a sequential process in which senescent leaves are subsequently abscised, resulting in lower observed levels of senescence during periods of increased leaf fall. This pattern highlights the interdependent nature of these phenophases within the plant’s physiological cycle. Since senescence and leaf fall represent sequential processes, the contrasting correlations likely reflect different stages within the same physiological response.

A negative correlation between fruiting and precipitation was also observed during the study period, suggesting that excess water may slow metabolism and inhibit nutrient allocation to fruiting, or represent a survival strategy in which fruiting occurs outside the rainy season to favor seed germination at the onset of rains [27]. This behavior has also been reported in species such as *Handroanthus heptaphyllus* (Vell.) Mattos [21], *Miconia nervosa* (Sm.) [28], and *Dipteryx odorata* (Aubl.) Forsyth f. [12], as well as in *Lippia dulcis* Trevir [15], highlighting that some species, particularly in tropical regions, tend to fruit during periods of lower rainfall.

Although some correlations were statistically significant, their biological interpretation should consider the moderately low correlation coefficients observed for certain relationships.

Overall, the data obtained reveal a complex relationship between the phenophases of *Lippia lasiocalycina* and abiotic factors. The analysis of phenophases and their correlation with environmental data suggested an association between these factors and the species’ phenology. These results suggest that the phenotypic adaptation of *L. lasiocalycina* to environmental conditions is fundamental for its survival and development, highlighting the importance of continuous monitoring to better understand the interactions between climatic and phenological variables. Such studies are essential for determining the appropriate timing for the collection of leaves, flowers, fruits, and seeds, as well as for understanding ecological interactions [12]. Although this study provides important initial insights into the phenological behavior of *Lippia lasiocalycina* under cultivation conditions in Feira de Santana, phenological patterns may vary among environments due to differences in climatic and edaphic conditions and may also differ from those of natural adult populations. In addition, because the study was conducted over a single annual cycle, interannual climatic variability was not assessed. Therefore, further long-term studies conducted across different locations are necessary to better understand the stability of and variability in the species’ phenological patterns under distinct environmental conditions.

## 4. Materials and Methods

### 4.1. Experimental Site

The study was conducted at the Experimental Unit of the Forest Garden (Horto Florestal) of the State University of Feira de Santana (UEFS), located at latitude −12.269588 and longitude −38.938432, in the municipality of Feira de Santana, approximately 116 km from Salvador, Bahia, Brazil. The plants were cultivated in a loam–clay–sandy soil, with a pH of 6.0, 30 mg dm^−3^ of phosphorus (P), 60 mg dm^−3^ of potassium (K), calcium levels of 3.9 cmolc dm^−3^, and magnesium levels of 1.1 cmolc dm^−3^. The soil had a base saturation (V%) of 79.68%, organic matter (OM) content of 1.23%, and a cation exchange capacity (CEC) of 6.59 cmolc dm^−3^.

### 4.2. Propagation and Cultivation

The biological material used in the study was obtained through vegetative propagation from different mother plants collected in Santa Terezinha, Bahia. These were deposited and identified in the university’s herbarium (HUEFS) under voucher number 19.3481, registered in SISGEN (Registration No. A1C1DEB), and maintained in the Medicinal and Aromatic Plant Collection of the UEFS Forest Garden. Plants were vegetatively propagated using cuttings collected from the apical and median regions of the branches, cultivated in 200 mL disposable cups filled with commercial Biomix^®^ substrate, and kept in mini-greenhouses covered with transparent plastic under intermittent irrigation. After 45 days, the plants were transferred to 1 L black polyethylene bags filled with agricultural soil and maintained in a greenhouse until final planting, when they had reached approximately 40 cm in height. The definitive planting was carried out in 30 cm × 30 cm × 30 cm pits filled with 1 L of well-composted cattle manure, with a spacing of 1 m between plants. A total of 24 plants were placed in the definitive site, where they remained in the field throughout the 12-month analysis period.

### 4.3. Phenology

Phenological data were collected at the end of the first fortnight and on the last day of each month from 24 plants over 12 consecutive months (January to December 2024), starting 90 days after planting, when all plants had reached reproductive activity. The following phenophases were identified: (1) budding: from the appearance of buds to the full development of new leaves; (2) mature leaves: fully developed and expanded leaves; (3) leaf senescence: indicated by leaf yellowing; (4) flowering: from the emergence of flower buds to the last mature flower; (5) fruiting: from the last mature flower to seed formation; (6) leaf fall: when fallen leaves and bare branches start to appear.

The intensity of phenological events was estimated for each plant [18] by employing a semi-quantitative interval scale with 5 categories: (0) absence of the phenophase, (1) phenophase present in 1–25% of the plant, (2) 26–50%, (3) 51–75%, and (4) 76–100%.

Data were analyzed using Fournier’s intensity percentage and the activity index (or percentage of individuals), with the latter representing only the presence or absence of each phenophase. The synchrony classification was based on the percentage of individuals expressing each phenophase, as determined by the activity index. The following thresholds were adopted to characterize synchrony levels: asynchronous (<20% of individuals), low synchrony (20–60%), and high synchrony (>60%). These categories were established according to the proportion of individuals simultaneously exhibiting the phenophase, allowing for the identification of phenological event synchrony within the population.

Meteorological data were obtained from the Brazilian National Institute of Meteorology (INMET) (https://portal.inmet.gov.br/), except for photoperiod data, which were derived from the geographic coordinates using the Astronomical Applications Department of the U.S. Naval Observatory (https://aa.usno.navy.mil/data/RS_OneYear accessed on 31 December 2024). The variables collected included total precipitation (mm), average temperatures (°C), photoperiod (h day^−1^), solar radiation (KJ m^−2^), and relative humidity (%). Meteorological variables were obtained as daily records and summarized according to the sampling interval of the phenological evaluations. Total precipitation was accumulated for each fortnightly period, while the rest were expressed as mean values for the same period.

### 4.4. Statistical Analysis

Seasonality of the phenophases was evaluated using circular statistics, in which the annual cycle was converted into 15° intervals corresponding to the biweekly evaluation dates. Circular analyses were performed based on the frequency of individuals expressing each phenophase at each sampling date. For each phenophase, the mean angle, mean date, vector length (r), and Rayleigh test significance were calculated in order to assess temporal concentration and seasonality throughout the annual cycle. Data normality was assessed using the Shapiro–Wilk test. Since the environmental variables, except relative humidity, did not meet normality assumptions, Spearman’s correlation coefficient was used to evaluate the relationships between climatic variables and phenophases. Correlation analyses were performed using fortnightly observations collected over 12 months, resulting in 24 paired observations. Statistical analyses were performed using R software version 4.2.3 (R Core Team, 2023).

## 5. Conclusions

The species exhibited high phenological synchrony for mature leaves and budding. Fruiting displayed seasonal patterns and showed a negative correlation with precipitation, with peak intensity occurring in the second half of November and the first half of December, which are the optimal periods for seed collection. The phenological pattern of the species under the environmental conditions of Feira de Santana, Bahia, Brazil, indicates the possibility of economic exploitation throughout all seasons of the year. This study highlights the importance of understanding the species’ phenological behavior, contributing to its conservation, cultivation, and sustainable management.

## Figures and Tables

**Table 1:** Results of circular statistics for the synchrony of phenological events, including budding, mature leaves, leaf senescence, flowering, leaf fall, and fruiting of *Lippia lasiocalycina* Cham. from January to December 2024 under the environmental conditions of Feira de Santana, Bahia, Brazil.

Phenophases	Average Angle	Average Date	r	*p*
Budding	Not defined	All year round	0	1
Mature leaves	Not defined	All year round	0	1
Leaf senescence	137.68	20/May	0.0178	0.8399
Flowering	158.61	10/Jun	0.057	0.283
Leaf fall	4.04	05/Jan	0.0469	0.319
Fruiting	147.78	30/May	0.179	0.0000028 *

* = Statistically significant (*p* < 0.05).

## Data Availability

Data are contained within the article. Further inquiries can be directed to the corresponding authors.
